# Comparative Whole-Genome Analysis of Russian Foodborne Multidrug-Resistant *Salmonella* Infantis Isolates

**DOI:** 10.3390/microorganisms10010089

**Published:** 2021-12-31

**Authors:** Anna Egorova, Yulia Mikhaylova, Stepan Saenko, Marina Tyumentseva, Aleksandr Tyumentsev, Konstantin Karbyshev, Aleksey Chernyshkov, Igor Manzeniuk, Vasiliy Akimkin, Andrey Shelenkov

**Affiliations:** Central Research Institute of Epidemiology, Novogireevskaya str., 3a, 111123 Moscow, Russia; mihailova@cmd.su (Y.M.); saenko@cmd.su (S.S.); tyumentseva@cmd.su (M.T.); tymencev@cmd.su (A.T.); karbyshev@cmd.su (K.K.); chernyshkov@cmd.su (A.C.); manzeniuk@cmd.su (I.M.); vgakimkin@yandex.ru (V.A.); fallandar@gmail.com (A.S.)

**Keywords:** *Salmonella* Infantis, antibiotic resistance, whole-genome sequencing, genomic epidemiology

## Abstract

Non-typhoidal *Salmonella* infections remain a significant public health problem worldwide. In this study, we present the first detailed genomic analysis report based on short-read (Illumina) whole-genome sequencing (WGS) of 45 multidrug-resistant (MDR) *Salmonella enterica* subsp. *enterica* serotype Infantis isolates from poultry and meat product samples obtained in Russia during 2018–2020, and long-read (MinION) WGS of five more representative isolates. We sought to determine whether foodborne *S.* Infantis have acquired new characteristics, traits, and dynamics in MDR growth in recent years. All sequenced isolates belonged to the sequence type ST32 and more than the half of isolates was characterized by six similar antimicrobial susceptibility profiles, most of which corresponded well with the antimicrobial resistance determinants to aminoglycosides, sulphonamides, tetracycline, and chloramphenicol revealed in silico. Some of the isolates were characterized by the presence of several types of plasmids simultaneously. Plasmid typing using WGS revealed Col440I, ColpVC, ColRNAI, IncFIB, IncFII, IncX1, IncHI2, IncHI2A, and IncN replicons. The identified virulence genes for 45 whole genomes of *S.* Infantis were similar and included 129 genes encoding structural components of the cell, factors responsible for successful invasion of the host, and secreted products. These data will be a valuable contribution to further comparative genomics of *S.* Infantis circulating in Russia, as well as to epidemiological surveillance of foodborne *Salmonella* isolates and investigations of *Salmonella* outbreaks.

## 1. Introduction

*Salmonella enterica* subsp. *enterica* is a Gram-negative bacterium that can be divided into two groups–typhoidal *Salmonella*, which are adapted for humans and do not occur in other animals, and non-typhoidal *Salmonella* (NTS), which usually cause foodborne gastroenteritis and can infect various animals. The severity of the disease depends on the host, the *Salmonella* serotype, and delivery method [1,2]. More than 2500 serotypes of NTS have been identified, however, only a limited number of them are responsible for the majority of salmonellosis cases reported [3,4,5].

The pathogenesis of these organisms depends on multiple virulence and antimicrobial resistance (AMR) genes [6], mostly associated with *Salmonella* pathogenicity islands (SPIs), prophages, and plasmids. Some virulence factors seem to be conserved among *Salmonella*, while others may be serotype-specific [7]. Most cases of *Salmonella*-caused diseases are mild, but sometimes they can be life threatening and, therefore, require appropriate antibiotic therapy. Antimicrobial resistance is a public health problem worldwide, and *Salmonella* is among the microorganisms with a number of resistant strains found in the food chain. Ciprofloxacin is a common first-line antimicrobial drug for the treatment of salmonellosis, but since fluoroquinolones are not used to treat children, beta-lactams (ampicillin or third-generation cephalosporins) are equally important [8]. MDR *S. enterica* serotypes are associated with higher morbidity outcomes compared to drug-susceptible strains [9].

Despite the fact that over 2500 *S. enterica* serotypes have been documented, most investigations were focused on *Salmonella enterica* subsp. *enterica* serotypes Typhimurium and Enteritidis. However, *S.* Infantis has emerged as the fourth most common serotype causing human salmonellosis in Europe [10]. According to the Russian Reference Center for Salmonellosis Monitoring, in recent years *S.* Infantis was one of the three predominant serotypes in Russia, along with *S.* Enteritidis and *S.* Typhimurium. Most often *S.* Infantis was revealed in food (28.4%) and in environmental samples (12%), while the frequency of isolation from human samples was much lower (1.5%) [11]. Since 2017, *S*. Infantis became the most common food origin serotype in Russia [12]. The growth of *S*. Infantis incidence has been registered in the period of 2003–2009 and still continues. [13,14]. *S*. Infantis seems to be particularly important in broiler, while the prevalence in laying hens and its products is much lower. The increase in *S.* Infantis carriage reported in broiler was observed in some EU/EEA countries, Israel, Turkey [15,16,17], and poultry and its products have been identified as one of the most important sources of human infection with *S.* Infantis [18]. On the other hand, analyzed *S.* Infantis samples from Turkey distributed between broilers and laying hens significantly unevenly—69% and 3% correspondingly [19]. Moreover, a similar picture we observed in Japanese research, where almost 100% of broiler isolates studied belonged to *S.* Infantis, and laying hens isolates did not contain them [20]. Increasing incidence and dissemination of different MDR *S*. Infantis lead to the problems in the public health system due to the treatment issues [21]. Therefore, there is a need to monitor the growth and occurrence of *S*. Infantis characteristics and the ways of its spreading in order to understand its newly acquired properties and provide better treatment options.

For public health surveillance systems, susceptibility data alone may not be sufficient in some circumstances, such as in assessment of resistant foodborne pathogens [22] and the risks associated with the use of agricultural antibiotics [23]. In risk analysis, it is often necessary to carry out additional genetic testing to compare alleles and genotypes of the isolates from different environments [24]. With the advent of whole-genome sequencing (WGS) technology, it is now possible to identify and evaluate the entire bacterial DNA sequence in just a few days, making it the best surveillance tool [25,26].

The WGS has been widely used in the field of molecular biology. However, the gaps between the fragments obtained by genome assembly from short reads can lead to the incompleteness of genome structure, which is critical for determining plasmid sequences and the location of particular genes [27]. Long-read nanopore sequencing technology can facilitate the completion of the bacterial genome assembly which either does not have sufficient sequencing depth in some of the repetitive regions or has areas with zero coverage when sequenced using short-read sequencing technology [28]. However, nanopore sequencing technology exhibits higher reading error frequencies and may produce systematic errors, and thus a combination of both technologies resulting in hybrid short and long-read assembly was used to obtain the accurate genomes of representative isolates in the current study.

The aim of this work is to present a comprehensive analysis of 45 MDR foodborne *S*. Infantis isolates from different regions of Russia based on WGS data to determine their characteristics and traits. In this paper, we provide data for *S.* Infantis isolates selected from 19 different geographical regions and belonging to one sequence type (ST) using short-read (Illumina) and long-read (MinION) WGS. The majority of the isolates (*n* = 26) were obtained from broiler and ready-to-cook meat products (*n* = 8). All these isolates were multidrug-resistant (MDR) according to the commonly used definition [29]. Plasmid structures, as well as antibiotic resistance, virulence genes and CRISPR elements’ annotation, are provided. We believe that these data will greatly facilitate the investigations of the distribution and the mechanisms of antimicrobial resistance acquisition for this pathogen.

## 2. Materials and Methods

### 2.1. Sample Isolation, Identification and Antibiotic Susceptibility Testing

Isolates from poultry product samples (*n* = 35) and meat product samples (*n* = 10) were obtained in the Russian Federation during a period of 2018-2020. Sample collection was carried out as a part of regular monitoring activities by 19 Russian Federal Centers of Hygiene and Epidemiology (Table 1). The highest number of the isolates were obtained in Krasnoyarsk (*n* = 6), Moscow and Moscow region (*n* = 6), Rostov (*n* = 5), and Perm (*n* = 4). The majority of the isolates (57.8%, *n* = 26) were obtained from chicken, 17.8% (*n* = 8) from ready-to-cook meat products, 15.6 % (*n* = 7) pertained to ready-to-cook chicken, while meat and turkey consisted of two samples each.

A total of 45 *Salmonella* isolates were identified down to a species level by time-of-flight mass spectrometry (MALDI-TOF MS) using the VITEK MS system (bioMerieux, Marcy-l’Étoile, France). The susceptibility of the samples included in this study was determined by the boundary concentration method on the same analyzer. Confirmation of the serotype Infantis was carried out manually using PETAL^®^ diagnostic *Salmonella* O-typing serum (Epidbiomed, Saint Petersburg, Russia). Antibiotic susceptibility testing (AST) of *S*. Infantis isolates was performed on the following antimicrobial panel: ampicillin (AMP), ampicillin/sulbactam (SAM), cefepime (FEP), ceftazidime (CZD/CAZ), cefuroxime axetil (CXM), ceftriaxone (CRO), cefoperazone/sulbactam (CFP), imipenem (IPM/M), ertapenem (ETP), ciprofloxacin (CIP), amikacin (AMK/AN/AKN), gentamicin (GMN/GEN), tobramycin (TOB), chloramphenicol (CHL), nitrofurantoin (NIT), trimethoprim/sulfamethoxazole (SXT), tetracycline (TET). The assessment of antimicrobial compounds included in this study was conducted according to the European Committee on Antimicrobial Susceptibility Testing interpretive standards (EUCAST). The results were interpreted according to EUCAST clinical breakpoints v.10.0 (http://www.eucast.org, accessed on 7 October 2021).

### 2.2. DNA Isolation and Whole Genome Sequencing

Genomic DNA was isolated with Ribo-prep Kit (Amplisens, Moscow, Russia) according to the manufacturer’s instructions. The quantity was evaluated by fluorimetry with Qubit 4.0 (Invitrogen, Waltham, MA, USA). DNA was used for paired-end library preparation with Nextera DNA Flex Library Prep Kit (Illumina, San Diego, CA, USA) according to the manufacturer’s recommendations. Short-read WGS was performed on the HiSeq 1500 platform (Illumina). Quality and quantity of libraries were confirmed by capillary gel electrophoresis (Agilent Bioanalyzer 2100, Agilent, Santa Clara, CA, USA) using a Qubit 4.0 fluorimeter (company, city, state abbreviation if USA, country).

Additionally, based on the initial analyses of short-read sequences, 5 more representative isolates were selected for long-read WGS for better characterization of plasmid structures. Libraries for long-read sequencing were prepared using the 1D Genomic DNA sequencing kit SQK-LSK108 (Oxford Nanopore, Oxford, UK). Size selection and clean-up steps were performed using AMPure XP (Beckman Coulter, Brea, CA, USA). Quantification of libraries was made using Qubit 4.0 (Thermo Fisher Scientific, Waltham, MA, USA). Sequencing of long reads was performed on MinION (Oxford Nanopore Technologies, Oxford, UK) R9 SpotON flow cell. Base calling of the raw MinION data was made using Guppy basecalling software version 4.4.1 (Oxford Nanopore Technologies) with default parameters, and demultiplexing was performed using Guppy barcoding software version 4.4.1 (Oxford Nanopore Technologies).

### 2.3. Genome Assembly and Sequence Analysis

Short-read genome assemblies were obtained using SPAdes version 3.12 [27] with default parameters. Hybrid short- and long-read assemblies were performed using Unicycler version 0.4.9 [28].

Assembly quality estimation, organism checking and initial annotation were performed using the custom pipeline described earlier [29]. We determined the antibiotic resistance genes in silico, performed isolate typing using multilocus sequence typing (MLST) scheme and revealed the presence of CRISPR/Cas systems and CRISPR arrays. The presence of virulence factors was also studied using the VFDB database (http://www.mgc.ac.cn/VFs/, accessed on 20 October 2021). *Salmonella* MLST website (https://enterobase.warwick.ac.uk/species/index/senterica, accessed on 20 October 2021) was used for sequence type detection. *Salmonella enterica* MLST schemes include the fragments of the following seven loci: *aroC*, *dnaN*, *hemD*, *hisD*, *purE*, *sucA*, and *thrA* [30]. We used the Resfinder 4.0 database with default parameters for antimicrobial gene identification [31]. Plasmid sequences were revealed and typed using PlasmidFinder with default parameters [32]. Plasmid images were built with SnapGene Viewer 6.0.0. CRISPRCasFinder with default parameters [31] was used to identify the presence of CRISPR/Cas systems and spacers in the genomes analyzed.

The assembled genome sequences for all isolates were uploaded to the NCBI Genbank under the project number PRJNA780779*Salmonella*.

To build the phylogenetic tree representing the relations between the isolates based on core genome sequence, we used roary version 3.13.0 [32] and RAxML version 8.2.11 [33] software with default parameters (model used: -m GTRGAMMA -p 13434345).

cgMLST analysis was performed using MentaList version 0.2.4 [34], and the minimum spanning tree was built using PHYLOViz online [33]. cgMLST scheme including 3002 targets was used (https://www.cgmlst.org/ncs/schema/schema/4792159/, accessed on 20 October 2021) [34].

### 2.4. CRISPR Typing and Analysis

CRISPRCasFinder [35] was used to identify the presence of CRISPR/Cas systems and spacers in the genomes studied.

*Salmonella* spacers were identified and downloaded from CRISPRFinder web tool [36] (http://crispr.i2bc.paris-saclay.fr/Server/, accessed on 20 October 2021), then FASTA sequences of the spacers were uploaded to Web BLAST^®^ blastn suite (https://blast.ncbi.nlm.nih.gov, accessed on 18 September 2021) and analyzed using default parameters of MegaBLAST algorithm.

Venn diagram was constructed using a free web service (https://bioinformatics.psb.ugent.be/webtools/Venn/, accessed on 20 October 2021).

## 3. Results

### 3.1. Isolation and Typing

The sources of foodborne *S.* Infantis isolates are described in Table 1. The majority of the isolates were obtained from Krasnoyarsk, Rostov, Moscow, and Perm Hygiene and Epidemiology Centers of Federal service on customers’ rights protection and human well-being surveillance (Rospotrebnadzor). Based on the MLST analysis, all isolates belong to the sequence type ST32.

**Figure 1:**
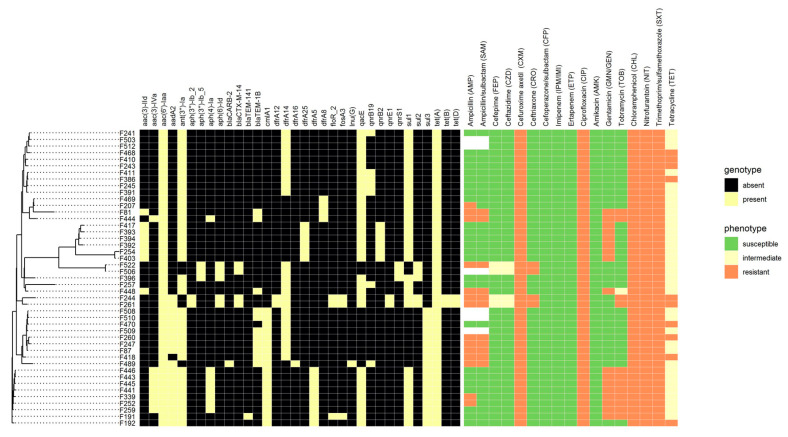
Phenotypic and genotypic antibiotic resistance profiles of the *S.* Infantis isolates studied. The tree shown on the left was built using ad hoc core genomes calculated by roary with the total number of core genes being 4371. White color means missing results for particular antibiotics.

In order to obtain additional information regarding bacteria similarity, a phylogenetic tree was built for all 45 isolates. The tree is presented below in Figure 1.

**Figure 2:**
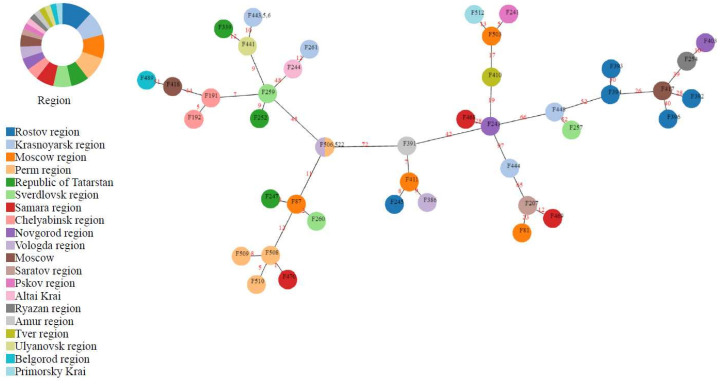
Minimum spanning tree (MST) based on cgMLST profiles for ST32 *S*. Infantis isolates. The number of different alleles between the profiles is shown on the branches connecting the respective isolates. The node CrieF443 had a completely identical profile to Crie-F445 and Crie-F446, thus they are combined in one node, as well as Crie-F522, whose profile was the same as for Crie-F506. Nodes are colored by the region of isolate collection.

According to the phylogenetic analysis, high similarity groups of the same sequence type (ST32) were revealed regardless of the different regions. For a deeper phylogenetic relationship analysis between the 45 *S.* Infantis isolates, a minimum spanning tree based on core genome MLST (cgMLST) was constructed (Figure 2). The clustering seems to be unrelated to the year of isolation, source, or geographic location. However, the resistance profile appears to have some influence. For instance, Crie-F87, Crie-F247, and Crie-F260 clustered together possessed the same antimicrobial susceptibility despite the fact that they were obtained from different regions. A similar picture was observed for the isolates Crie-F244 and Crie-F261; and, in addition, for Crie-F441, Crie-F443, Crie-F445, Crie-F446, and Crie-F339.

### 3.2. Antibiotic Resistance Determination

WGS technology currently allows detecting the presence of genes, but cannot reveal their expression or activation state, thus, a comprehensive comparison of empirical and theoretical susceptibility determination is required. A total of 45 *S*. Infantis isolates were tested for antibiotic susceptibility/resistance both by empirical (boundary concentration) method and bioinformatic analysis (searching for known acquired resistance genes in genomic sequences). A comprehensive comparison of empirical and theoretical susceptibility determination is presented in Figure 1.

All 45 isolates were resistant to six antimicrobial drugs out of 17 tested. The boundary concentration method identified resistance to CXM, CIP, CHL, NIT, SXT, and TET, which belong to different classes of antibiotics. Two isolates (Crie-F244 and Crie-F261) were resistant to 12 antimicrobial drugs, one (Crie-F522) to 11 antibiotics, and three isolates (Crie-F81, Crie-F444, Crie-F448) to 10 antibiotics from the panel used.

Moreover, with a help of WGS (Figure 1) we revealed that the main spectrum of antimicrobial resistance determinants included genes of aminoglycoside-modifying enzymes *aac(6′)-Iaa* and *ant(3″)-Ia* (45 and 40 of the isolates, respectively), as well as genes involved in tetracycline (*tetABD*, *n* = 45), and sulfonamide (*sul123*, *dfrA14*, *n* = 45) resistance that corresponds well to AST. However, the presence of AMR genes to chloramphenicol, quinolones, and β-lactams did not correspond well with the phenotypic profiles. For example, *cmlA1* gene had 18 isolates and *floR* had three samples, while the susceptibility test indicated that all isolates studied possessed a resistance to CHL (Figure 2). Moreover, all isolates observed had resistance to CIP and NIT, although AMR genes for fluoroquinolones (*qnrB19* and *qnrB2*) were revealed only in 13 isolates, and 5 isolates included quinolone AMR genes (*qnrE1* and *qnrS1*) (Figure 2). The most common mechanism for resistance to quinolones in *Salmonella* is the acquisition of point mutations in chromosomal AMR gene *gyrA* [37]. As we did not check isolates for point mutations and intrinsic chromosomal AMR genes, we can suggest that the resistance was associated with substitutions at the Ser-83 position to Tyr, Phe, or Ala, or/and Asp-87 substitutions to Asn, Gly, or Tyr [37]. Therefore the molecular mechanism of quinolone resistance will possibly be clarified in further reports by point mutation analysis.

Interestingly, the susceptibility test identified resistance to the second generation cephalosporins (CXM) throughout the isolates studied, and just four isolates (Crie-F244, Crie-F261, Crie-F506, and Crie-F522) possessed a resistance to the third (CZD and CRO) and fourth (FEP) generation cephalosporins. At the same time, according to the AMR profile only these four isolates contain *blaCTX-M-14* (Figure 2). Thus, we confirm that *blaCTX-M-14* infuse resistance to 3rd and 4th generation cephalosporins in *S*. Infantis as has been previously described [14].

### 3.3. Plasmids

Since plasmids play a key role in the acquisition of antimicrobial resistance, it is important to identify plasmid sequences in the WGS data, as this information will greatly facilitate the study of resistance mechanisms. The results of in silico search for the plasmids in all genomic sequences of the isolates are presented in Table 2 below. The most common plasmid replicon groups in our collection included a range of plasmids previously associated with multidrug-resistant foodborne bacteria IncFIB (*n*  =  42) and widely distributed IncX1 (*n* = 13) previously detected in *Salmonella*, *Klebsiella*, *Shigella*, and *E. coli* species [35,36]. Some of the isolates were characterized by the presence of several plasmid types simultaneously (Crie-F386, Crie-F503, and Crie-F510). IncX1 plasmids containing a group of *qnr* genes (resistance to quinolones) are considered widespread [35,36].

Five isolates, Crie-F191, Crie-F247, Crie-F252, Crie-F410, and Crie-F444, had different MDR phenotypes and were resistant to eight, eight, nine, six and ten antimicrobial compounds, respectively (Figure 1). These MDR isolates were subjected to long-read WGS using the Oxford Nanopore device.

In-depth genomic analysis by hybrid assembly confirmed that five MDR isolates carried pESI-like megaplasmids with resistance and virulence gene patterns previously detected in *S.* Infantis from Russia. According to the WGS analysis, all samples except Crie-F191 included the IncFIB replicon, while three isolates had additional IncX1 in another plasmid. Crie-F191 had an IncFII replicon, however, hybrid assembly included the same megaplasmid as in Crie-F252 with IncFIB (Table 2, Table S1). Their size ranged from 285 kb to approximately 317 kb (Table S1). The samples Crie-F247 and Crie-F444 contained an additional plasmid of approximately 39 kb length previously found in *Escherichia coli* according to BLAST analysis of ‘nt’ database.

**Figure 3:**
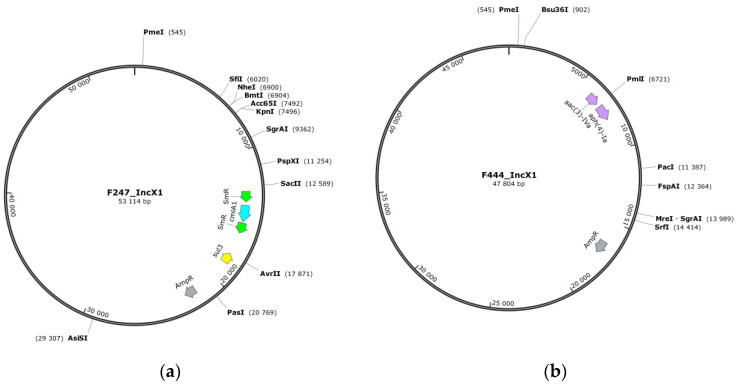

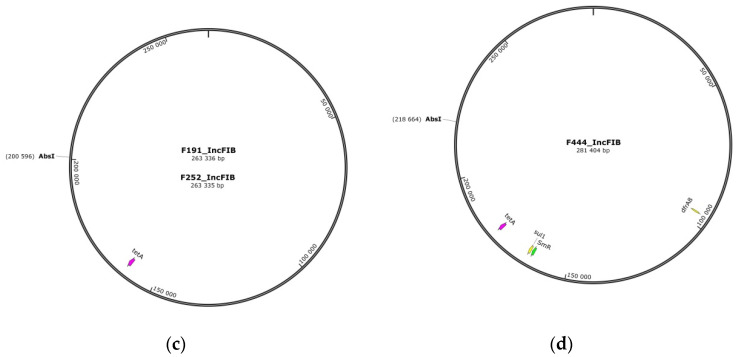
Plasmid replicons IncX1 and IncFIB determined by hybrid assembly. (**a**) Small IncX1plasmid (53 kb) harbored by Crie-F247 *S*. Infantis isolate. (**b**) IncX1plasmid (47 kb) located in Crie-F444 *S*. Infantis isolate. (**c**) IncFIB plasmid (263 kb) harbored by Crie-F191 and Crie-F252. (**d**) IncFIB plasmid (281 kb) from Crie-F444. Resistance to doxycycline and tetracycline is highlighted with pink color, resistance to SXT—with yellow, resistance to streptomycin—with green, chloramphenicol AMR genes are shown in blue, ampicillin resistance—in gray, and AMR to aminoglycosides is highlighted with purple. The plasmids were visualized via SnapGene, annotation was performed manually.

Plasmids reconstructed in silico by the hybrid assembly are presented below in Figure 3 using SnapGene tool. Visual modeling allowed us to reveal significant differences in IncX1 replicons from our samples (Figure 3a,b), while IncFIB appeared to be more similar between the isolates. For instance, plasmids of Crie-F191 and Crie-F252 contain single resistance gene *tetA* and have almost the same size (Figure 3c). On the other hand, IncFIB replicon of Crie-F247 has one additional *dfrA14* resistance gene to SXT, and, at the same time, IncFIB replicon of Crie-F444 has also gained resistance gene to SXT (Figure S1), but in this case the gene is *sul1* and it is located in different position. Moreover, Crie-F444 contains AMR gene to streptomycin (Figure 3d). It seems that IncFIB initially contained *tetA* genes and others could be transferred from other plasmids or microorganisms.

The pESI-like plasmids were very similar across the isolates studied: IncFIB carried virulence genes, while IncX1 carried beta-lactamase and some other resistance genes, with one to eight resistance genes in each isolate (Figure 3). All five of these isolates included a *tet(A)* gene, while Crie-F247, and Crie-F444 additionally had *ant(3″)-Ia* and *blaTEM-1B*. In total, we observed 12 antimicrobial resistance genes associated with pESI-like plasmids (*aadA2*, *blaTEM-1B*, *dfrA14*, *dfrA8*, *sul1*, *sul3*, *tetA*, *cmlA1*, *ant(3″)-Ia*, *qacE*, *aac(3)-IVa*, *aph(4)-Ia*).

The megaplasmids analyzed carried specific virulence gene pattern ESIv (Emergent *S.* Infantis virulence) [18] which consists of the genes encoding for the virulent yersiniabactin operon (*fyuA*, *IRP1*, *irp2*, *ybtAEPQSTUX*) and the genes associated with the fimbrial protein synthesis (*faeCDEFHI*).

### 3.4. Virulence Genes

The pathogenic potential of bacteria is determined by multiple factors which affect the complex interactions of *Salmonella* with the host [3]. These genes could be located on plasmids or within the chromosome in a form of one or several virulence genes, or large cassettes composed of a series of genes and operons (SPIs) [38]. *Salmonella* has five major known SPIs involved in different pathways from cell invasion and survival to the systemic phase of infection and enteropathogenesis [14,39,40].

The set of virulence determinants in the isolates studied was identical and quite extensive (up to 129). According to Figure S2, genes of fimbrial and non-fimbrial adherence gene, type III secretion system (SPI-1 and SPI-2) involved in host invasion and intracellular bacteria replication, respectively, SPI-3 required for intracellular survival or for virulence, and a gene providing resistance to antimicrobial peptides were identified. Moreover, SPI-5 which encoded effectors that were induced by distinct regulatory pathways (*sopB* and *pipB*) were observed. All 129 found virulence genes were present in all ST32 isolates, except for one gene in a sample Crie-F244 (*sipD*), which occurs in SPI-1 type III secretion system. The main virulence factors observed are given in Figure S2. A list of all 129 virulence determinants is provided in Table S2.

### 3.5. CRISPR/Cas Systems

CRISPR/Cas system might provide effective information useful for the isolate typing since *Salmonella* spp. serotypes carry two CRISPR loci and their motifs differ in length [41,42]. Therefore, CRISPR arrays were analyzed. All isolates carried CRISPR loci with encoded Cas proteins, which are an important component for the functioning of the putative CRISPR/Cas system, and such systems belonged to Type IE. Furthermore, all ST32 isolates included in this study contained 2, 3, or 4 CRISPRs motifs. They were formed of 29 bp, 39 bp, and 38 bp long repeats except for Crie-F417, Crie-F392 that had 23 bp length repeats, and Crie-F261 with a 51 bp repeat. We assumed that bioinformatic assay was responsible for the inconsistency in the detection of repeat length. Analysis of CRISPR arrays in *S.* Infantis isolates studied revealed 2432 spacers. Among them, 2424 were repeating (with 117 unique among them) and only eight spacers were unique. Unique spacers were found in CRISPR arrays of Crie-F207 (five spacers out of eight), Crie-F261 (one out of eight), Crie-F417 (one out of eight), and Crie-F468 (one out of eight) isolates (Table S3).

**Figure 4:**
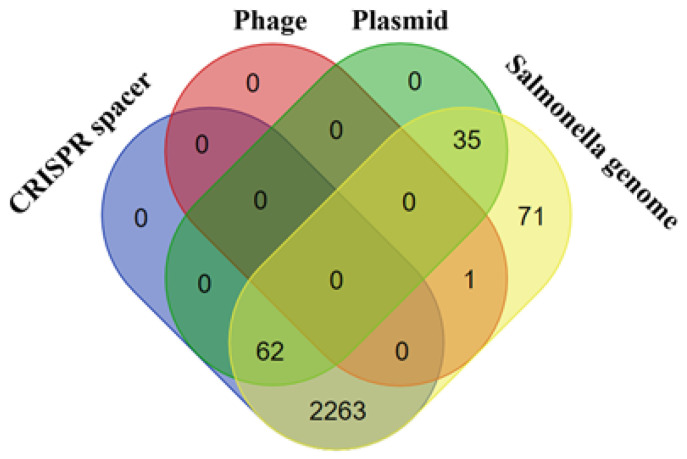
Homology of CRISPR spacers for the *Salmonella* isolates studied.

The vast majority (2325 of 2432) of spacers were identified by BLAST (using the megablast algorithm) as *Salmonella* CRISPR spacers (e.g., ‘*Salmonella enterica* subsp. *enterica* serotype Infantis strain 03-2387 CRISPR1 repeat region’). Only 0.04% of spacers (one spacer out of 2432) were identified by BLAST (using the megablast algorithm) as phage sequences (e.g., ‘*Enterobacteria phage* mEpX2, complete genome’), 4% of spacers—as plasmid sequences (e.g., ‘*Escherichia coli* isolate MSB1_4I-sc-2280412 genome assembly, plasmid: 11′) and none of the spacers—as pathogenicity island encoding sequences (Figure 4, Table S3). Interestingly, two repetitive spacers (one from Crie-F191; Crie-F192; Crie-F252; Crie-F339; Crie-F445; Crie-F446, others—from Crie-F259 and Crie-F443 CRISPR array)—were not identified by BLAST using the megablast algorithm. Using the blastn algorithm both spacers were denoted to *Salmonella enterica* (Table S3).

Notably, CRISPR arrays located on the one side of *cas* cassette from 18 out of 45 isolates were identical. On the other side of *cas* cassette CRISPR arrays (positions 1-152 bp) of these isolates formed two separate groups of arrays. Similarly, CRISPR arrays located on one side of *cas* cassette from 26 *Salmonella* isolates were identical and also formed two separate groups of arrays on the other side of *cas* cassette. One part of CRISPR arrays from Crie-F468 and Crie-F469 shared homology with the second group of *Salmonella* isolates. The other one formed a separate group of CRISPR arrays with partial identity.

The CRISPR/Cas loci of the 45 isolates were predicted. Interestingly, in all isolates except one putative cassettes had the same composition with some discrepancies in the combination of the *cas* cassette position. The loci from all of the samples except Crie-F243 were identical and consisted of eight genes encoding Cas1 endonuclease, Cas2 endoribonuclease, Cas3 helicase, and Cas5, Cas6, Cas7, Cse1, and Cse2 proteins (Table S4). At the same time, Crie-F243 did not have Cse2, and it seems that this deletion has led to *cas* cassette shifting (Table S4). Moreover, all systems could be grouped into two clusters: the first had a sequence of CRISPR-associated (*cas*) genes *cas2*, *cas1*, *cas6*, *cas5*, *cas7*, *cse2*, *cse1*, *cas3*, while the second was *cas3*, *cse1*, *cse2*, *cas7*, *cas5*, *cas6*, *cas1*, *cas2* (Table S4). On the other hand, there is no evidence that the position of *cas* genes is of some functional significance.

## 4. Discussion

The *S. enterica* serotype Infantis is widespread not only in poultry, especially broilers, but also in pigs and cattle [43]. However, specifically, poultry and its products have been identified as one of the most important sources of salmonellosis infection in humans [44]. In the last few years, antimicrobial resistance has emerged in *S.* Infantis from various food sources and humans worldwide [44,45]. Thus, the increased dissemination of various MDR *S.* Infantis clones has led to the spreading of organisms in the food chain and through poultry to people in such countries as Turkey [21], Switzerland [9], Germany [18], and Russia [14]. For this reason, we aimed to find out whether the *S.* Infantis isolates of food origin collected in 2018−2020 have acquired new properties which resulted in changes of the pathogen characteristics and to reveal possible dynamics in the MDR development over the past few years.

In the current report, we analyzed 45 MDR *S. enterica* isolates obtained from various food products in 19 different geographical regions of Russia during the period of 2018–2020 within the framework of food poisoning monitoring by the regional centers of Hygiene and Epidemiology. All isolates studied belonged to ST32 and Infantis serotype, which was the most prevalent in Europe, the Middle East, and Japan [9,18,46]. Moreover, susceptibility testing showed that all of them were resistant to six antimicrobial drugs: CXM, CIP, CHL, NIT, SXT, and TET. Ciprofloxacin and β-lactams are common first-line antibiotics used for treating salmonellosis [8]. As described in the Hindermann et al., resistance to ciprofloxacin was verified in 4.6% of the isolates from Switzerland [9], while almost all isolates studied in Germany were resistant to CIP and TET [18]. Thus, we can suggest that there could be a problem with CIP therapy in Russia since our collection of ST32 isolates obtained in 2018–2020 possessed a resistance to this antibiotic according to the susceptibility testing. Moreover, the majority of the isolates from Switzerland showed a combined resistance to SXT and TET, and some samples were resistant to ampicillin [9], which was the same for our samples.

The major AMR determinants detected by WGS in the isolates studied included the genes of aminoglycoside-modifying enzymes, and genes responsible for resistance to tetracycline, sulphonamide, and chloramphenicol. Most of these genes were described in previous studies [7,18,22,47]. For example, the chromosomally encoded gene *aac(6′)-Iaa* was detected among all the *S.* Infantis samples of food origin obtained in different countries [7,18]. Various studies have shown that phenotypic resistance to tetracycline was strongly correlated with the presence of known resistance determinants predicted by the WGS with the most frequent being the tetracycline efflux transporter encoded by *tet(A)* [22,47], while sulfonamides resistance was predominantly encoded by *sul1* and *sul2* [47], which conforms well with our observations. For instance, all foodborne *S.* Infantis isolates from Turkey possessed *tetA* and *sul1* genes in combination and carried *aac(6′)-Iaa* and *ant(3″)-Ia* genes [21] encoding aminoglycoside modifying enzymes, just as our samples described above. However, most of the isolates were sensitive to aminoglycoside tested drugs (gentamicin, tobramycin) in the current study. At the same time, all our isolates included AMR gene pattern specific for the MDR strains revealed in Germany in 2010, which consisted of *ant(3″)-Ia*, *tet(A)*, and *sul1* [18].

The current study also showed that the antimicrobial resistance phenotypes and genotypes correlated well in general, however, some discrepancies were found. This can be explained by the fact that the presence of a resistance gene in a genome cannot be considered as single evidence for its expression and activity in the given isolate, which is determined by a complex of factors [48]. For example, discrepancies were observed in low presence of β-lactam (*bla* family) and quinolone (*qnr*) genes, while the susceptibility test showed widespread resistance to some antibiotics of these classes. Interestingly, all isolates studied possessed CXM resistance and were quite susceptible to third and fourth-generation cephalosporins (Figure 1). It can be explained that third and fourth-generation cephalosporins have specific modifications to penetrate through the outer membrane of Gram-negative bacteria. However, we found β-lactamase AMR genes that could made *S.* Infantis resistant to CRO, CZD, and FEP. Surprisingly, according to the data from the USA, *dfrA12* and *dfrA14* genes were unique to the human isolates of *Salmonella* [22], while our foodborne samples carried both these resistance genes, and thus we may suggest that they could be transferred from humans to food products via manual handling. However, the data from Turkey showed similarity to Russian genotypes of *S*. Infantis isolates in a sense that more than half of Turkish isolates had *dfrA14* [21]. Moreover, WGS results revealed that three out of 45 *S.* Infantis isolates harbored the *floR* gene, for which disagreement of phenotypic resistance to chloramphenicol was observed, while foodborne samples from Turkey showed resistance to chloramphenicol/florfenicol without the presence of the *floR* gene [21]. In our case, phenotypic resistance of some isolates could be explained by *cmlA1* occurrence. However, the possible resistance mechanism requires a more detailed study. AMR phenotypes and genotypes information is important for epidemiological studies and accumulation of general data on resistance genes circulating in the bacterial phavingopulation.

Short-read sequencing indicated the presence of IncFIB replicon, which belongs to the family of IncF, in 93.3% **S*. Infantis* isolates. IncF plasmids are widely distributed in the *Enterobacteriaceae* family and carry virulence factors, AMR, cytotoxins, and adhesion factors [49], which were confirmed by the presence of the main traits of resistance and virulence found in the German isolates of *S.* Infantis which were positive for the IncFIB replicon [18]. Moreover, for example, IncF replicon families have been associated with the sudden worldwide emergence of resistance to quinolones and aminoglycosides due to the presence of *aac(6′)-Ib-cr*, which was absent in our AMR profile, and *qnr* genes [36,49]. Resistance to aminoglycosides in our isolates was possibly encoded by the *aac(6′)-Iaa* gene revealed in all of them, which differs from the resistance genes usually present on the replicons of IncF plasmids [49]. Thus we can assume that the aac*(6′)-Iaa* gene can be located on the chromosome. As previously described, it was supposed that ampicillin resistance can be carried not only by plasmids belonging to the incompatibility group I1 (IncI1) [50,51], but also by other plasmid families, such as the small plasmids Col and IncX possessing *blaTEM* resistance genes [52,53]. The genes *ant(3″)-Ia*, *sul1*, *sul3*, *tetA* were found by long-read sequencing in pESI-like plasmids. Interestingly, *sul* genes and *ant(3″)-Ia* presented in our study were located in different incompatibility replicons, while replicon typing identified the IncF incompatibility group that carried *sul* genes (*sul1*, *sul2*, and *sul3*) in *E. coli* isolates of food origin from China [54]. At the same time, we suggest that *ant(3″)-Ia* could be presented in both megaplasmids and small plasmids as described by Kürekci et al. [21]. Moreover, the IncFIB replicons contain *tet(A)*, *sul1* and *dfr* A14 genes which represent widespread elements of *S.* Infantis [14,21].

Whereas the majority of genes for full virulence are found in the chromosome as SPIs [38], we analyzed 129 virulence genes harbored by all *S.* Infantis isolates of our sample collection (Table S2). All 45 samples showed the same virulence profile regardless of the year, geographical region or source of isolation (Figure S2), except for one gene in a sample Crie-F244 (*sipD*). However, this is most likely a sequencing inaccuracy, since Crie-F261 has the same profile of the AMR genes and plasmids, and included the whole spectrum of virulence genes presented. The fimbrial adherence gene operons *bcf*, *fim*, *inv*, *csg*, the type III secretion system 1 (T3SS-1) genes: *sipABC*, *sopABE2* and *spaOPQRS*, the type III secretion system 2 (T3SS-2) genes: *spiC*, *sifA*, *sscAB*, *sse*, and *ssa* operons were similar to the most common strains in Tunisia [7]. Type III secretion systems (SPI-1 and SPI-2) were identified among the set of virulence determinants in the isolates studied. SPIs located on plasmids or in chromosome consist of multiple genes for full virulence and might have been acquired by horizontal transfer from phage or plasmids of other organisms [38,55]. Additionally, they are highly conserved between the different *Salmonella* serotypes [38]. A single SPI can turn a normally non-hazardous microorganism into a pathogen, but the acquisition of SPI cannot ensure such a transformation [56].

In addition, we detected the *S.* Infantis plasmid-encoded fimbria *lpf* operon and genes encoding for the virulent yersiniabactin operon (*fyuA*, *irp1*, *irp2*, *ybt*), which were associated with the specific gene pattern ESIv (Emergent *S.* Infantis virulence) [18,57]. As previously reported [14], similar resistance and virulence gene patterns have been determined by the presence of a pESI-like plasmid. Thus, we decided to perform genomic detection and further characterization of pESI-like plasmids among our most representative isolates. ESIv of *S.* Infantis isolates studied consists of the genes encoding for the virulent yersiniabactin operon (*fyuA*, *irp1*, *irp2*, *ybtAEPQSTUX*) and genes associated with the fimbrial protein synthesis (*faeCDEFHI*). *S.* Infantis from Turkey displayed the presence of fimbrial operons such as *Agf/Csg*, *fim*, and *lpf*, and many of them were located on pESI-like plasmid, as well as YbtTU and Irp1 regulatory and transport operons of *Yersinia pestis* [21]. Interestingly, the yersiniabactin biosynthetic protein-coding cassette from *Klebsiella pneumoniae* was located in the chromosome, as mentioned earlier [58].

Notably, all *S.* Infantis isolates studied carried CRISPR/Cas systems belonging to Type IE. In our study, the spacers targeting *S. enterica* genomes were also analyzed. According to CRISPRminer Self-targeting (http://www.microbiome-bigdata.com/CRISPRminer/index.php/Home/Index/selfTarget accessed on 13 May 2021) 870 of 22110 self-targeting spacers belong to *S. enterica*. Among the 71 spacers which were targeting *S. enterica* genomes (Table S3) one spacer was unique and 70 were repeating (with five unique ones among repeating spacers). On the one hand, it is known that CRISPR/Cas systems can acquire self-targeting spacers from the host chromosome which results in autoimmunity and cell death [59], but, on the other hand, such spacers are suggested to be involved in mRNA degradation that allows evading immune detection [60]. CRSIPR/Cas systems possessing self-targeting spacers may require tight regulation to properly balance the danger of autoimmunity with the risk of phage infection, and thus deserve further investigation [60]. Taking into account all of the above, we can assume that CRISPR sequences are serotype-specific and, thus CRISPR/Cas system might provide the information useful for effective typing [41,42,61].

## 5. Conclusions

In the current report we presented a comprehensive analysis of 45 MDR foodborne *S.* Infantis isolates from different regions of Russia based on WGS data including molecular typing, antibiotic resistance profiling, virulence factors, and plasmid descriptions. Notably, Russia is not a member of the PulseNet International (PulseNet Europe) surveillance system, that probably may be one of the reasons of an insufficiency in genetic diversity data of foodborne *Salmonella* serotypes [62]. Moreover, the few studies carried out in Russia are mainly limited to the observation of strains isolated from salmonellosis outbreaks [62]. In addition, we found only one confirmation of WGS analysis for foodborne *Salmonella* [14]. Thus, to the best of our knowledge, our WGS-based study is the most extensive in terms of the number of the isolates in Russia in general, and of a single serotype in particular. Additionally, the long-read sequencing was performed for the first time for such isolates, which allowed getting some insights into the mechanisms of antibiotic resistance transmission.

We believe that the data obtained will greatly facilitate further epidemiological surveillance of *S.* Infantis as an important foodborne pathogen in terms of genome dynamics and monitoring the newly acquired resistance and virulence characteristics.

## Figures and Tables

**Table 1:** The metadata for the foodborne *S.* Infantis isolates studied.

Isolate	Year Collected	Region	Source
Crie-F81	2018	Moscow region	Chicken
Crie-F87	2018	Moscow region	Chicken
Crie-F191	2019	Chelyabinsk region	Ready-to-cook chicken
Crie-F192	2019	Chelyabinsk region	Chicken
Crie-F207	2019	Saratov region	Turkey
Crie-F241	2019	Pskov region	Ready-to-cook chicken cutlets
Crie-F243	2019	Novgorod region	Meat
Crie-F244	2019	Altai Krai	Chicken
Crie-F245	2019	Rostov region	Ready-to-cook meat
Crie-F247	2019	Republic of Tatarstan	Ready-to-cook meat
Crie-F252	2019	Republic of Tatarstan	Chicken
Crie-F254	2019	Ryazan region	Ready-to-cook chicken cutlets
Crie-F257	2019	Sverdlovsk region	Ready-to-cook meat cutlets
Crie-F259	2019	Sverdlovsk region	Chicken
Crie-F260	2019	Sverdlovsk region	Ready-to-cook meat
Crie-F261	2019	Krasnoyarsk region	Ready-to-cook meat cutlets
Crie-F339	2019	Republic of Tatarstan	Chicken
Crie-F386	2019	Vologda region	Ready-to-cook meat cutlets
Crie-F391	2019	Amur region	Ready-to-cook chicken cutlets
Crie-F392	2019	Rostov region	Chicken
Crie-F393	2019	Rostov region	Chicken
Crie-F394	2019	Rostov region	Ready-to-cook chicken cutlets
Crie-F396	2019	Rostov region	Chicken
Crie-F403	2019	Novgorod region	Chicken
Crie-F410	2019	Tver region	Ready-to-cook chicken cutlets
Crie-F411	2019	Moscow region	Chicken
Crie-F417	2019	Moscow	Chicken
Crie-F418	2019	Moscow	Chicken
Crie-F441	2020	Ulyanovsk region	Chicken
Crie-F443	2020	Krasnoyarsk region	Chicken
Crie-F444	2020	Krasnoyarsk region	Chicken
Crie-F445	2020	Krasnoyarsk region	Chicken
Crie-F446	2020	Krasnoyarsk region	Chicken
Crie-F448	2020	Krasnoyarsk region	Chicken
Crie-F468	2020	Samara region	Turkey
Crie-F469	2020	Samara region	Pork
Crie-F470	2020	Samara region	Ready-to-cook meat
Crie-F489	2020	Belgorod region	Chicken
Crie-F503	2020	Moscow region	Ready-to-cook beef cutlets
Crie-F506	2020	Perm region	Ready-to-cook chicken cutlets
Crie-F508	2020	Perm region	Chicken
Crie-F509	2020	Perm region	Chicken
Crie-F510	2020	Perm region	Chicken
Crie-F512	2020	Primorsky Krai	Chicken
Crie-F522	2020	Vologda region	Chicken

**Table 2:** Plasmid replicons found in the isolates.

Isolate	Plasmid Replicon			
	Col	IncF	IncX	Other
Crie-F81	-	IncFIB	IncX1	-
Crie-F87	-	IncFIB	IncX1	-
Crie-F191	-	IncFII	-	-
Crie-F192	-	-	-	-
Crie-F207	Col440I	-	-	-
Crie-F241	-	IncFIB	-	-
Crie-F243	-	IncFIB	-	-
Crie-F244	-	IncFIB	-	IncHI2, IncHI2A, IncN
Crie-F245	-	IncFIB	-	-
Crie-F247	-	IncFIB	IncX1	-
Crie-F252	-	IncFIB	-	-
Crie-F254	-	IncFIB	-	-
Crie-F257	-	IncFIB	-	-
Crie-F259	-	IncFIB	-	-
Crie-F260	-	IncFIB	IncX1	-
Crie-F261	-	IncFIB	-	IncHI2A, IncHI2, IncN
Crie-F339	-	IncFIB	-	-
Crie-F386	ColpVC	IncFIB	-	-
Crie-F391	-	IncFIB	-	-
Crie-F392	-	IncFIB	-	-
Crie-F393	-	IncFIB	-	-
Crie-F394	-	IncFIB	-	-
Crie-F396	-	IncFIB	-	-
Crie-F403	-	IncFIB	-	-
Crie-F410	-	IncFIB	IncX1	-
Crie-F411	-	IncFIB	-	-
Crie-F417	-	IncFIB	-	-
Crie-F418	-	IncFIB	IncX1	-
Crie-F441	-	IncFIB	-	-
Crie-F443	-	IncFIB	-	-
Crie-F444	-	IncFIB	IncX1	-
Crie-F445	-	IncFIB	-	-
Crie-F446	-	IncFIB	-	-
Crie-F448	-	IncFIB	IncX1	-
Crie-F468	-	IncFIB	IncX4	-
Crie-F469	-	IncFIB	-	-
Crie-F470	-	IncFIB	IncX1	-
Crie-F489	-	IncFIB	IncX1	-
Crie-F503	ColRNAI, ColpVC	IncFIB	-	-
Crie-F506	-	IncFIB	-	-
Crie-F508	-	IncFIB	IncX1	-
Crie-F509	-	IncFIB	IncX1	-
Crie-F510	Col(pHAD28), ColRNAI	IncFIB	IncX1	-
Crie-F512	-	IncFIB	-	-
Crie-F522	-	IncFIB	-	-

## Data Availability

The assembled genome sequences for all isolates were uploaded to the NCBI Genbank, the project number PRJNA780779, under the following accession numbers: JAJMUC000000000 (Crie-F81), JAJJZC000000000 (Crie-F87), JAJMUB000000000 (Crie-F191), JAJJZB000000000 (Crie-F192), JAJJZA000000000 (Crie-F207), JAJMUA0000 F2400000 (Crie-F244), JAJMTX000000000 (Crie-F245), JAJMTW000000000 (Crie-F247), JAJMTV000000000 (Crie-F252), JAJMTU000000000 (Crie-F254), JAJMTT000000000 (J250000A F257) Crie-F260), JAJMTQ000000000 (Crie-F261), JAJMTP000000000 (Crie-F339), JAJMTO000000000 (Crie-F386), JAJMTN000000000 (Crie-F391), JAJJYZ000000000 (JrieMJTM000000000 (CrieAJTM000000) -F394), JAJMTL000000000 (Crie-F396), JAJMTK000000000 (Crie-F403), JAJMTJ000000000 (Crie-F410), JAJMTI000000000 (Crie-F411), JAJMTH000000000 (Crie-FM4000000) F441), JAJMTF000000000 (Crie-F443), JAJMTE000000000 (Crie-F444), JAJMTD000000000 (Crie-F445), JAJMTC000000000 (Crie-F446), JAJJYW000000000 (Crie-F448000000) JAJ (Crie-F468), JAJMTA000000000 (Crie-F469), JAJMSZ000000000 (Crie-F470), JAJMSY000000000 (Crie-F489), JAJJYV000000000 (Crie-F503), JAJMSX000000000 (Crie-F503), JAJMSX000000000 (Crie-C000000000), JAMSV000000 Crie-F509), JAJMSU000000000 (Crie-F510), JAJJYU000000000 (Crie-F512), JAJJYT000000000 (Crie-F522) *Salmonella*.

## Supplementary Materials

The following are available online at www.mdpi.com/article/10.3390/microorganisms10010089/s1, Figure S1: Plasmid replicon IncFIB (270 kb) harbored by Crie-F247 *S*. Infantis isolate determined by hybrid assembly, Figure S2: Virulence genes presence for the isolates studied revealed by bioinformatics methods. The number of representative genes was selected specific to *S. enterica*, Table S1: in silico megaplasmid data provided by hybrid assembly, Table S2: List of 129 virulence genes observed by VFDB database. Table S3: Spacers of *S*. Infantis isolates studied found using blastn algorithm, Table S4: Data providing by CRISPRCasFinder. Group of similar genes is shown by one color.
