# Supplementary material for: Comparative Whole-Genome Analysis of Russian Foodborne Multidrug-Resistant Salmonella Infantis Isolates
Source: Microorganisms. 2021 Dec 31;10(1):89. doi: 10.3390/microorganisms10010089 (PMC8781764; doi:10.3390/microorganisms10010089)
Supplement: Supplementary file 1 [file microorganisms-10-00089-s001.zip › Supplementary Information.pdf]

Supplementary Information for

Comparative Whole-Genome Analysis of Russian Foodborne Multidrug-Resistant *Salmonella Infantis* Isolates

Anna Egorova*, Yulia Mikhaylova, Stepan Saenko, Marina Tyumentseva, Aleksandr Tyumentsev, Konstantin Karbyshev, Aleksey Chernyshkov, Igor Manzenuk, Vasiliy Akimkin and Andrey Shelenkov

Central Research Institute of Epidemiology, Novogireevskaya str., 3a, 111123 Moscow, Russia

bioanna1995@gmail.com; mihailova@cmd.su (Y.M.); saenko@cmd.su (S.S.); tyumentseva@cmd.su (M.T.); tymencev@cmd.su; karbyshev@cmd.su; chernyshkov@cmd.su (A.C.); manzeniuk@cmd.su (I.M.); vgakimkin@yandex.ru (V.A.); fallandar@gmail.com (A.S.)

* Correspondence: bioanna1995@gmail.com

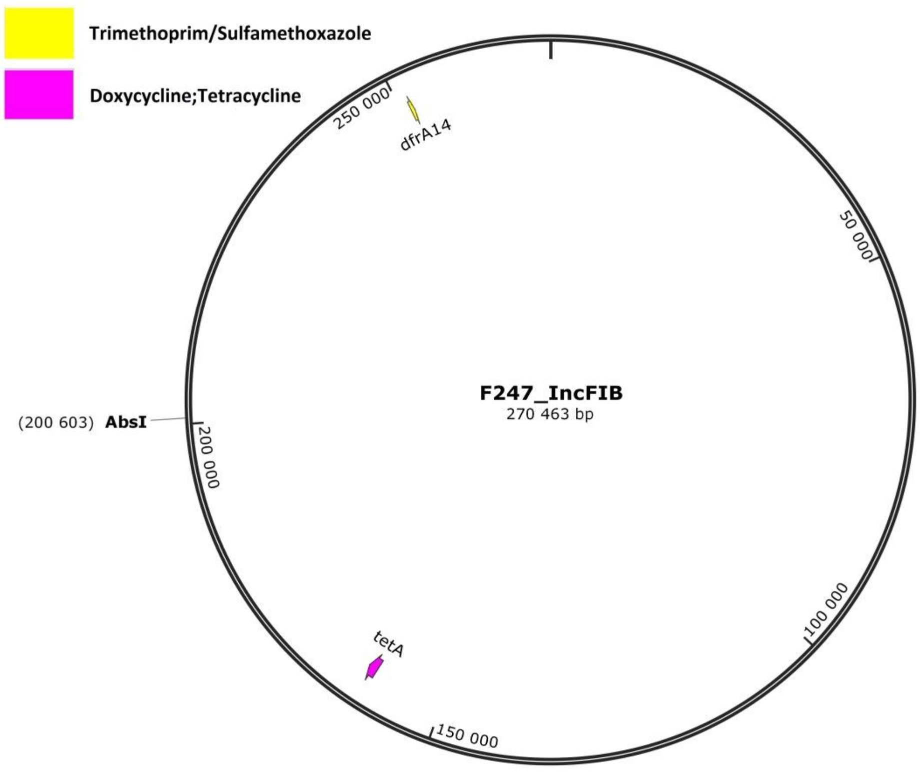

Figure S1. Plasmid replicon IncFIB (270 kb) harbored by Crie-F247 *S*. Infantis isolate determined by hybrid assembly

Sample	Salmonella virulence factors																				
	fimCDFHI	lpfABCDE	invAEFGHIJ	orgA/sctK	prgHIJK	sicAP	sipBC	sipD	spaOPQRS	pipB	sifAB	sopD2	spiC/ssaB	sseABCDFGJ	sspH2	mgtBC	misL	mig-14	ratB	shdA	sinH
F191	+	+	+	+	+	+	+	+	+	+	+	+	+	+	+	+	+	+	+	+	+
F192	+	+	+	+	+	+	+	+	+	+	+	+	+	+	+	+	+	+	+	+	+
F207	+	+	+	+	+	+	+	+	+	+	+	+	+	+	+	+	+	+	+	+	+
F241	+	+	+	+	+	+	+	+	+	+	+	+	+	+	+	+	+	+	+	+	+
F243	+	+	+	+	+	+	+	+	+	+	+	+	+	+	+	+	+	+	+	+	+
F244	+	+	+	+	+	+	+	-	+	+	+	+	+	+	+	+	+	+	+	+	+
F245	+	+	+	+	+	+	+	+	+	+	+	+	+	+	+	+	+	+	+	+	+
F247	+	+	+	+	+	+	+	+	+	+	+	+	+	+	+	+	+	+	+	+	+
F252	+	+	+	+	+	+	+	+	+	+	+	+	+	+	+	+	+	+	+	+	+
F254	+	+	+	+	+	+	+	+	+	+	+	+	+	+	+	+	+	+	+	+	+
F257	+	+	+	+	+	+	+	+	+	+	+	+	+	+	+	+	+	+	+	+	+
F259	+	+	+	+	+	+	+	+	+	+	+	+	+	+	+	+	+	+	+	+	+
F260	+	+	+	+	+	+	+	+	+	+	+	+	+	+	+	+	+	+	+	+	+
F261	+	+	+	+	+	+	+	+	+	+	+	+	+	+	+	+	+	+	+	+	+
F339	+	+	+	+	+	+	+	+	+	+	+	+	+	+	+	+	+	+	+	+	+
F386	+	+	+	+	+	+	+	+	+	+	+	+	+	+	+	+	+	+	+	+	+
F391	+	+	+	+	+	+	+	+	+	+	+	+	+	+	+	+	+	+	+	+	+
F392	+	+	+	+	+	+	+	+	+	+	+	+	+	+	+	+	+	+	+	+	+
F393	+	+	+	+	+	+	+	+	+	+	+	+	+	+	+	+	+	+	+	+	+
F394	+	+	+	+	+	+	+	+	+	+	+	+	+	+	+	+	+	+	+	+	+
F396	+	+	+	+	+	+	+	+	+	+	+	+	+	+	+	+	+	+	+	+	+
F403	+	+	+	+	+	+	+	+	+	+	+	+	+	+	+	+	+	+	+	+	+
F410	+	+	+	+	+	+	+	+	+	+	+	+	+	+	+	+	+	+	+	+	+
F411	+	+	+	+	+	+	+	+	+	+	+	+	+	+	+	+	+	+	+	+	+
F417	+	+	+	+	+	+	+	+	+	+	+	+	+	+	+	+	+	+	+	+	+
F418	+	+	+	+	+	+	+	+	+	+	+	+	+	+	+	+	+	+	+	+	+
F441	+	+	+	+	+	+	+	+	+	+	+	+	+	+	+	+	+	+	+	+	+
F443	+	+	+	+	+	+	+	+	+	+	+	+	+	+	+	+	+	+	+	+	+
F444	+	+	+	+	+	+	+	+	+	+	+	+	+	+	+	+	+	+	+	+	+
F445	+	+	+	+	+	+	+	+	+	+	+	+	+	+	+	+	+	+	+	+	+
F446	+	+	+	+	+	+	+	+	+	+	+	+	+	+	+	+	+	+	+	+	+
F448	+	+	+	+	+	+	+	+	+	+	+	+	+	+	+	+	+	+	+	+	+
F468	+	+	+	+	+	+	+	+	+	+	+	+	+	+	+	+	+	+	+	+	+
F469	+	+	+	+	+	+	+	+	+	+	+	+	+	+	+	+	+	+	+	+	+
F470	+	+	+	+	+	+	+	+	+	+	+	+	+	+	+	+	+	+	+	+	+
F489	+	+	+	+	+	+	+	+	+	+	+	+	+	+	+	+	+	+	+	+	+
F503	+	+	+	+	+	+	+	+	+	+	+	+	+	+	+	+	+	+	+	+	+
F506	+	+	+	+	+	+	+	+	+	+	+	+	+	+	+	+	+	+	+	+	+
F508	+	+	+	+	+	+	+	+	+	+	+	+	+	+	+	+	+	+	+	+	+
F509	+	+	+	+	+	+	+	+	+	+	+	+	+	+	+	+	+	+	+	+	+
F510	+	+	+	+	+	+	+	+	+	+	+	+	+	+	+	+	+	+	+	+	+
F512	+	+	+	+	+	+	+	+	+	+	+	+	+	+	+	+	+	+	+	+	+
F522	+	+	+	+	+	+	+	+	+	+	+	+	+	+	+	+	+	+	+	+	+
F81	+	+	+	+	+	+	+	+	+	+	+	+	+	+	+	+	+	+	+	+	+
F87	+	+	+	+	+	+	+	+	+	+	+	+	+	+	+	+	+	+	+	+	+

- involved in fimbrial adherence
- SPI-1 of Type III secretion system
- SPI-2 of Type III secretion system
- SPI-3 of Type III secretion system
- Resistance to antimicrobial peptides
- involved in nonfimbrial adherence

Figure S2. Virulence genes presence for the isolates studied revealed by bioinformatics methods. The number of representative genes was selected specific to *S.enterica*.
