# Supplementary figures and images for: Comparative Whole-Genome Analysis of Russian Foodborne Multidrug-Resistant Salmonella Infantis Isolates
Source: Microorganisms. 2021 Dec 31;10(1):89. doi: 10.3390/microorganisms10010089 (PMC8781764; doi:10.3390/microorganisms10010089)

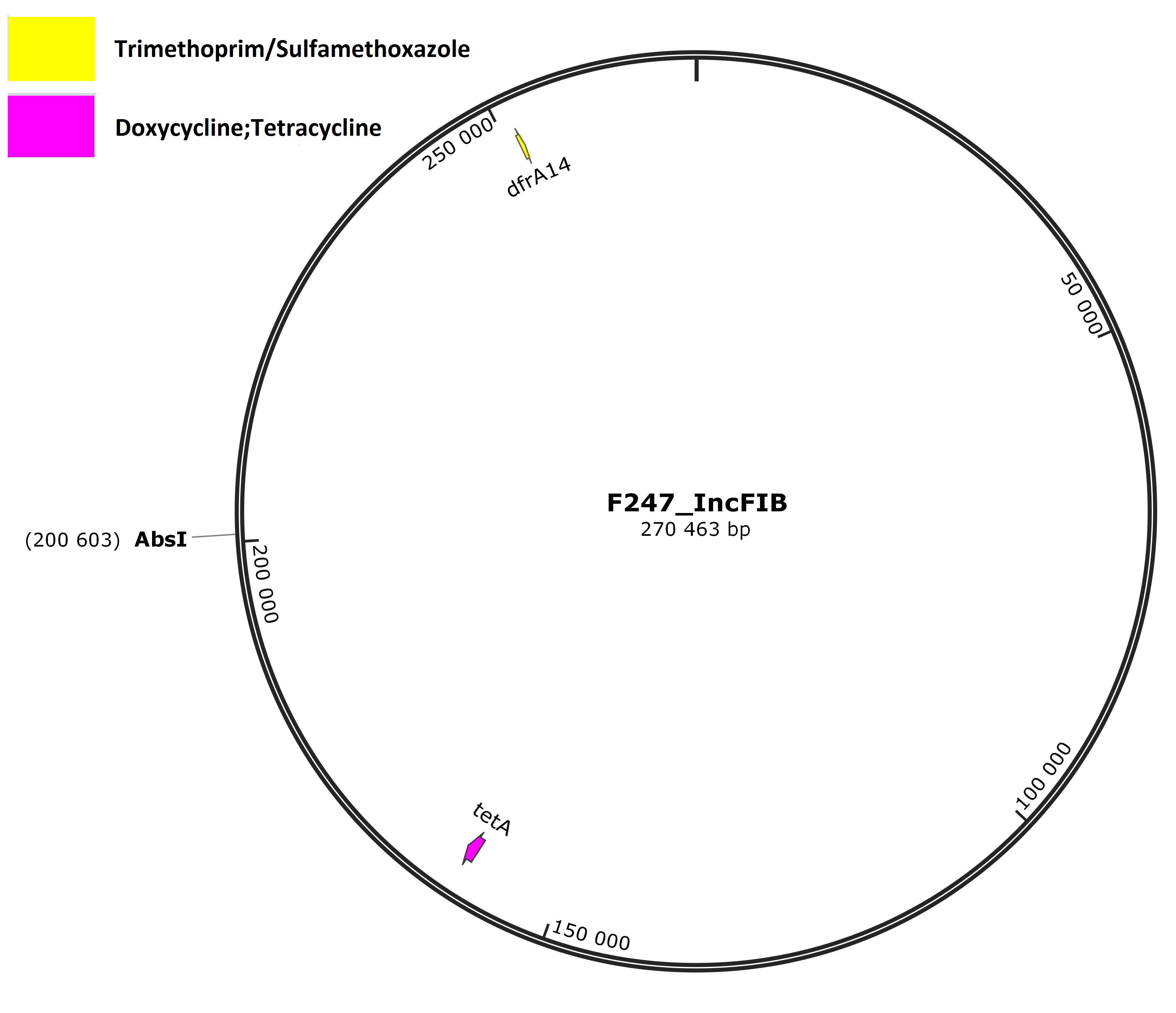

Supplement: Supplementary file 1 [file microorganisms-10-00089-s001.zip › figure S1.jpg]

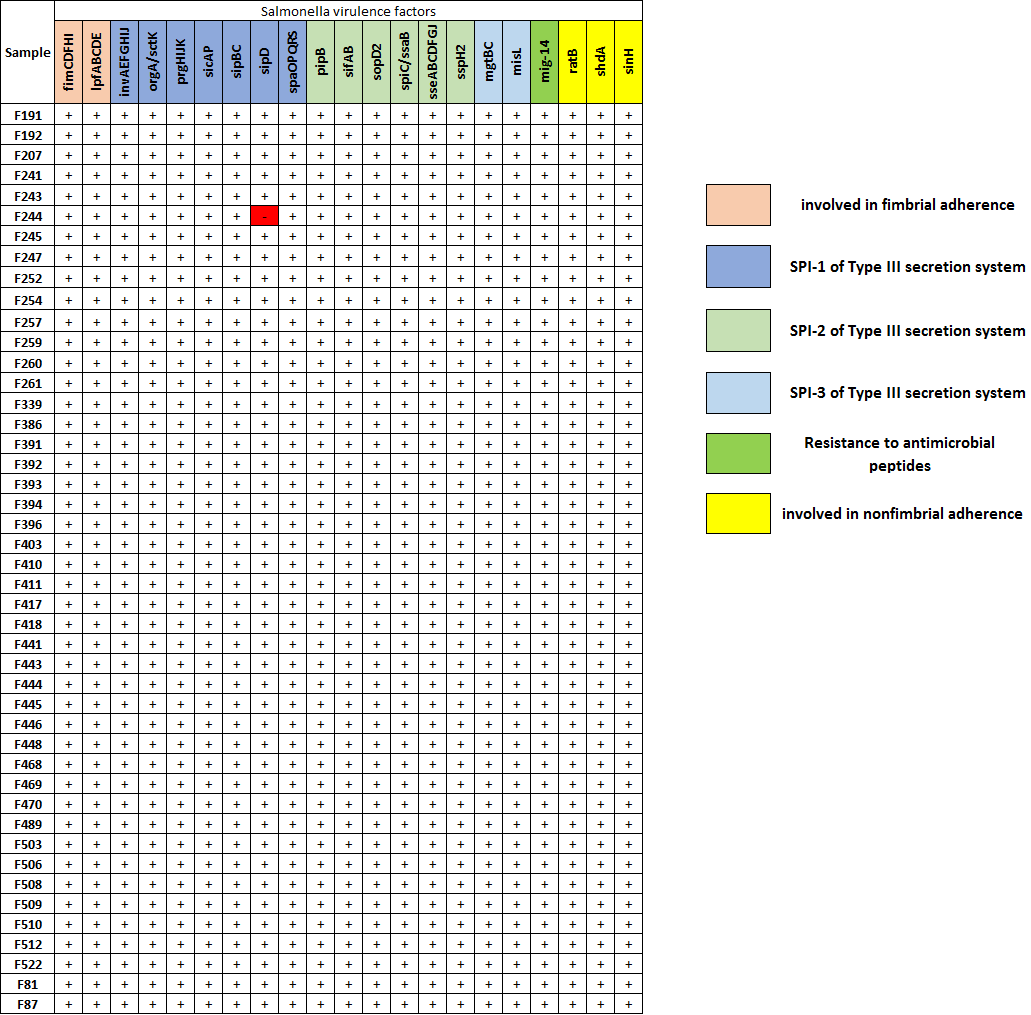

Supplement: Supplementary file 1 [file microorganisms-10-00089-s001.zip › figure S2.png]
